# Isolation and Characterization of Genes Responsible for Naphthalene Degradation from Thermophilic Naphthalene Degrader, *Geobacillus* sp. JF8

**DOI:** 10.3390/microorganisms8010044

**Published:** 2019-12-24

**Authors:** Daisuke Miyazawa, Le Thi Ha Thanh, Akio Tani, Masaki Shintani, Nguyen Hoang Loc, Takashi Hatta, Kazuhide Kimbara

**Affiliations:** 1 Institute of Plant Science and Resources, Okayama University, 2-20-1 Chuo, Kurashiki, Okayama 710-0046, Japan; Daisuke.Miyazawa@mitsuichemicals.com (D.M.); atani@okayama-u.ac.jp (A.T.); 2 Department of Environment and Energy System, Graduate School of Science and Technology, Shizuoka University, 3-5-1 Johoku, Naka-ku, Hamamatsu, Shizuoka 432-8011, Japan; hathanh116@gmail.com; 3 Institute of Bioactive Compounds, University of Sciences, Hue University, Hue, Thua Thien Hue 530000, Vietnam; nhloc@hueuni.edu.vn; 4 Department of Bioscience, Graduate School of Science and Technology, Shizuoka University, 3-5-1 Johoku, Naka-ku, Hamamatsu, Shizuoka 432-8561, Japan; 5 Department of Engineering, Graduate School of Integrated Science and Technology, Shizuoka University, 3-5-1 Johoku, Naka-ku, Hamamatsu, Shizuoka 432-8561, Japan; 6 Research Institute of Green Science and Technology, Shizuoka University, 836 Ohya, Suruga-ku, Shizuoka, Shizuoka 422-8529, Japan; 7 Department of Biomedical Engineering, Okayama University of Science, 1-1 Ridai-cho, Kita-ku, Okayama 703-8232, Japan; thatta@bme.ous.ac.jp

**Keywords:** naphthalene, *Geobacillus*, thermophile

## Abstract

*Geobacillus* sp. JF8 is a thermophilic biphenyl and naphthalene degrader. To identify the naphthalene degradation genes, *cis*-naphthalene dihydrodiol dehydrogenase was purified from naphthalene-grown cells, and its N-terminal amino acid sequence was determined. Using a DNA probe encoding the N-terminal region of the dehydrogenase, a 10-kb DNA fragment was isolated. Upstream of *nahB*, a gene for dehydrogenase, there were two open reading frames which were designated as *nahAc* and *nahAd*, respectively. The products of *nahAc* and *nahAd* were predicted to be alpha and beta subunit of ring-hydroxylating dioxygenases, respectively. Phylogenetic analysis of amino acid sequences of NahB indicated that it did not belong to the *cis*-dihydrodiol dehydrogenase group that includes those of classical naphthalene degradation pathways. Downstream of *nahB*, four open reading frames were found, and their products were predicted as *meta*-cleavage product hydrolase, monooxygenase, dehydrogenase, and gentisate 1,2-dioxygenase, respectively. A reverse transcriptase-PCR analysis showed that transcription of *nahAcAd* was induced by naphthalene. These findings indicate that we successfully identified genes involved in the upper pathway of naphthalene degradation from a thermophilic bacterium.

## 1. Introduction

**Figure 1:**
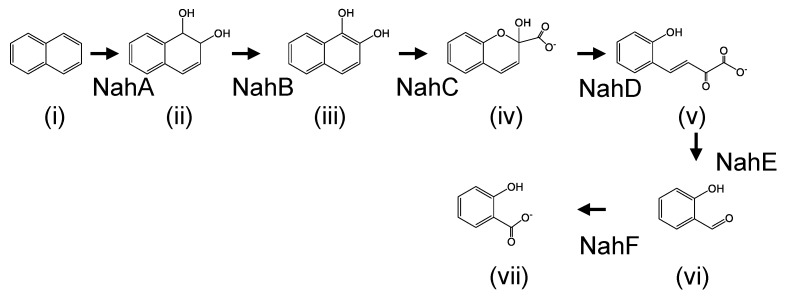
Naphthalene degradation pathway in mesophilic degraders. (**i**) naphthalene, (**ii**) *cis*-1,2-Dihydroxy-1,2-dihydronaphthalene, (**iii**)1,2-dihydroxynaphthalene, (**iv**) 2-hydroxychromene-2-carboxylate, (**v**) trans-*o*-hydroxybenzylidenepyruvate, (**vi**) salicylaldehyde, (**vii**) salicylate. NahA, naphthalene 1,2-dioxygenase; NahB, 1,2-dihydroxy-1,2-dihydronaphthalene dehydrogenase; NahC, 1,2-dihydroxynaphthalene dioxygenase; NahD, 2-hydroxychromene-2-carboxylate isomerase; NahE, *trans*-*o*-hydroxybenzylidenepyruvate hydratase-aldolase; NahF, salicylaldehyde dehydrogenase.

Naphthalene degradation pathways and their enzymes have been extensively studied in mesophilic bacteria including *Pseudomonas* species. Their degradation genes are organized in three operons: one of them encodes the enzymes involved in conversion of naphthalene to salicylate (naphthalene-degradation upper pathway); the second operon encodes the enzymes for conversion of salicylate to tricarboxylic acid cycle intermediates (pyruvate and acetyl-CoA) through the *meta*-cleavage pathway (naphthalene-degradation lower pathway); the third operon encodes a positive transcriptional regulator (NahR) [1,2,3,4,5,6,7]. The initial step of naphthalene degradation is the addition of dioxygen to naphthalene by naphthalene 1,2-dioxygenase (NahA enzyme), and it is converted to *cis*-1,2-dihydroxy-1,2-dihydronaphthalene (DDNP) (Figure 1). Secondly, DDNP is dehydrated and converted to 1,2-dihydroxynaphthalene (DHNP) by 1,2-dihydroxy-1,2-dihydronaphthalene dehydrogenase (NahB enzyme) (Figure 1). Thirdly, another dioxygen is added to DHNP by 1,2-dihydroxynaphthalene dioxygenase (NahC enzyme) and the DHNP is cleaved at the *meta* position on the benzene ring (Figure 1). The *meta*-cleavage product was converted to salicylate with some steps (Figure 1). The salicylate could be degraded via catechol or gentisate [1,8,9].

In contrast, thermophilic naphthalene-degrading bacteria were rarely reported [10,11,12,13]. *Geobacillus thermoleovorans* Hamburg 2 degrades naphthalene through a different pathway from that known in mesophilic naphthalene degraders [11]. Previously, we isolated a thermophilic naphthalene-degrader, *Geobacillus* sp. JF8 [10] and successfully identified its 1,2-dihydroxynaphthalne dioxygenase (NahC) from the cell of JF8 grown with naphthalene [14]. The amino acid sequences of NahC exhibited only 20–22% identity to 1,2-dihydroxynaphthalene dioxygenase of those of mesophiles (NahC of *Pseudomonas putida* G7, NahC of *Pseudomonas stutzeri* AN10, or NagC of *Ralstonia* sp. U2). Currently, no genes were found around the above *nahC*_JF8 showing similarity to the genes for the naphthalene-degradation upper pathway.

In the present study, we successfully purified a dihydrodiol dehydrogenase of JF8 and identified genes for the naphthalene-degradation upper pathway.

## 2. Materials and Methods

### 2.1. Bacterial Strains and Culture Conditions

*Geobacillus* sp. JF8 was grown at 60 °C on Castenholtz D solid medium (1.5% agar). Naphthalene was provided as vapor. The composition of Castenholtz D medium has been described previously [8]. *pahAc*_OUS82 and *pahAd*_OUS82 (encoding terminal dioxygenase of naphthalene dioxygenase from *Pseudomonas putida* OUS82), a kind gift from Prof. H. Kiyohara of Okayama University of Science, were on a 2.6-kb XhoI fragment in pHSG396 [15]. *Escherichia coli* JM109, used for construction and maintenance of plasmids, was cultured at 37 °C on LB medium [16]. Ampicillin (100 μg/mL), chloramphenicol (30 μg/mL), isopropyl -β-d-galactopyranoside (IPTG) and 5-bromo-4-chloro-3-indolyl-β-d-galactopyranoside (X-Gal) was used for selection of plasmids.

### 2.2. Southern Blot Analyses

A DNA probe spanning the determined N-terminal region of the dihydrodiol dehydrogenase was prepared by PCR with the degenerate primers, DDD-F; 5′-GARAAYAARGTiGCiTTYAT-3′ and DDD-R; 5′-ATiGCYTGYTTiACYTGYTC-3′ (R: A or G; Y: C or T, i: inosine). The probe was labeled with digoxygenin by DIG DNA Labeling and Detection Kit (Roche Diagnostics GmbH, Mannhiem, Germany). Total DNA of *Geobacillus* sp. JF8 was digested with BamHI, EcoRI, HindIII, SmaI, and XbaI, respectively, and the resultant DNAs were subjected to electrophoresis in 0.7% agarose gel before transfer to Hybond-N+ nylon membrane (GE Healthcare Life Sciences, Buckinghamshire HP7 9NA, England). For colony hybridization, the recombinant *E. coli* colonies were transferred to Hybond N+ nylon membranes. Southern hybridization was performed under high stringency as previously described [14].

### 2.3. Cloning and DNA Sequencing

Two DNA libraries were constructed with 9-kb SmaI or 5-kb EcoRI fragments of the chromosomal DNA of *Geobacillus* sp. JF8 in Charomid 9-36 (Nippon Gene Co. Ltd., Tokyo, Japan). The positive clones of *E. coli* with the above-mentioned DIG-probe, containing pCBEI5 (with 5-kb EcoRI fragment) and pCBSm9 (with 9-kb SmaI fragment) were obtained by colony hybridization. A 6-kb SacI-SmaI fragment was subcloned into pUC18 (Takara Bio Inc., Shiga, Japan) from pBSm9 and designated as pBSS6. Similarly, the 5-kb EcoRI fragment of pCBEI5 was subcloned into pSTV28 (Takara) designated as pBEI5. Nucleotide sequences of their inserts were determined as follows. First, nested unidirectional deletions in the target DNA, the 5 kb EcoRI and 6 kb SacI/SmaI inserts were created by the Kilo-sequence Deletion kit (Takara). Then, the nucleotide sequences of the different deleted inserts were determined using the CEQ DTCS-Quick Start Kit (Beckman Coulter Inc. Brea, CA, USA) by the dideoxy-chain termination method, with a CEQ2000 DNA sequencer (Beckman Coulter Inc.). DNA regions upstream and downstream of the cloned 7.6-kb fragment were isolated by genome walking. To obtain the downstream DNA fragment, a 0.5-kb BamHI-EcoRI fragment was used as a probe of Southern hybridization with BamHI-digested total DNA. A 4 kb BamHI fragment was isolated.

### 2.4. Enzymatic Assays

Enzymatic activity of NahB_JF8 was estimated by following the reduction of NAD^+^ using a DU-650 Spectrophotometer (Beckman Coulter Inc.) at 30 °C [17]. The extinction coefficients used for NADH were λ_max_ = 340 nm, ε = 6.22/μM/cm. Reaction mixtures contained 50 mM phosphate buffer (pH7.5), 30 μM *cis*-naphthalene dihydrodiol, 2.4 mM NAD+, 1 mM ascorbate, and enzyme solution. One enzyme unit was defined as the amount of enzyme required to reduce 1.0 μmol NAD^+^ per min.

### 2.5. Purification of the Cis-Dihydrodiol Dehydrogenase

*Geobacillus* sp. JF8 was grown on Castenholz D agar plates in the presence of naphthalene vapors. The cells were harvested, washed and resuspended in 20 mM phosphate buffer (pH 7.5) containing 30 mM β-mercaptoethanol and 0.5 mM EDTA (Buffer A). The cell suspension was passed through a French Press (Thermo Fisher Scientific Inc. Tokyo, Japan) and centrifuged at 17,000× *g* for 60 min. The supernatant was applied to a DEAE-Toyopearl 650M (Tosoh) column. The enzyme was eluted with 4 L gradient of 0.0 to 0.5 M KCl. The eluted protein was dialyzed and applied to a Phenyl sepharose FF 16/10 column (GE Healthcare Life Sciences) equilibrated with Buffer A containing 0.1 M ammonium sulfate (Buffer B). The enzyme was eluted with a 400 mL gradient of 1.0 to 0.0 M ammonium sulfate. The eluted protein was dialyzed against 20 mM phosphate buffer (pH7.0) containing 30 mM β-mercaptoethanol, 0.5 mM EDTA, and 0.2 M ammonium sulfate (Buffer B2). Then the sample was applied to a Phenyl sepharose FF 16/10 column equilibrated with Buffer B2. The enzyme fraction was eluted by a 200 mL gradient of 1.0 to 0.0 M ammonium sulfate, and the enzyme was dialyzed against 20 mM phosphate buffer (pH7.0) containing 30 mM β-mercaptoethanol and 0.5 mM EDTA (Buffer A2) and water. The samples eluted by Buffer A2 and water were combined dialyzed against 20 mM Tris-HCl buffer (pH8.5) containing 30 mM β-mercaptoethanol and 2 mM EDTA (Buffer C). Finally, the resultant samples were applied to a Mono Q HR 5/5 column (GE Healthcare Life Sciences) equilibrated with Buffer C. The enzyme was eluted with a 40 mL gradient of 0.0 to 0.5 M KCl. The fractions containing enzyme activity were pooled and dialyzed against Buffer A. The N-terminal sequence of the native enzyme was determined by automated Edman degradation on a Model 492 Protein Sequencer (Thermo Fisher Scientific Inc.).

### 2.6. Electrophoresis

Sodium dodecyl sulfate-polyacrylamide gel electrophoresis (SDS-PAGE) was carried out according to the method of Laemmli [18] with Prestained Protein Marker Broad Range (New England Biolabs Japan Inc., Tokyo, Japan). Native nondenaturing PAGE was performed with HMW Native Marker Kit (GE Healthcare Life Sciences) using the same solutions without SDS. Gels were stained with Coomassie Brilliant Blue R250 [16]. The relative molecular masses were calculated from the mobilities of the marker proteins.

### 2.7. RNA Extraction and RT-PCR

**Figure 2:**
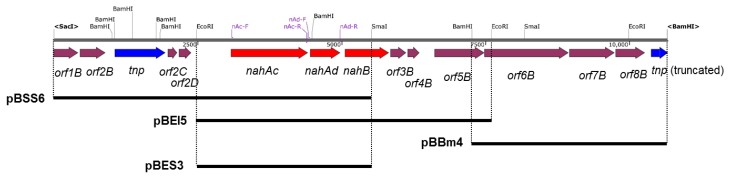
Genetic organization of the nah gene cluster of strain JF8. Red, blue, and magenta pentagons indicate naphthalene-degradative genes, transposase gene, and genes for hypothetical proteins. Restriction enzyme site and positions for primers are shown. The solid black bars under the map indicate DNA regions cloned into each plasmid.

*Geobacillus* sp. JF8 was grown on LB plate at 60 °C for 8 h, then the resultant colonies were exposed to naphthalene vapor for 4 h to induce their degradative pathways. Total RNA was extracted from the JF8 cells using a RNeasy Mini Kit (Qiagen, Venlo, The Netherlands). The RNA samples were treated with DNase I (Thermo Fisher Scientific Inc). Reverse transcriptase (RT)-PCR was carried out with 1-ng RNA of each sample and OneStep RT-PCR kit (Qiagen) using primers; nAc-F: 5′-AGAACAAATCGAAGGCGTTT-3′ and nAc-R: 5′-TTTCCATTGACGCCAAATG-3′, and nAd-F: 5′-TGAGCTTTCGATACCGAGACA-3′ and nAd-R: 5′-ACCGCTAAGTTATCCATCCCT -3′. The primer sets of nAc-F and nAc-R were used to amplify *nahAc* gene and those of nAd-F and nAd-R were for *nahAd* gene (Figure 2). The conditions for the RT-PCR amplification were as follows; 50 °C for 30 min; 95 °C for 15 min; followed by 35 cycles of 94 °C for 30 s, 48 °C for 1 min, and 72 °C for 5 min. For negative controls, the initial incubation at 50 °C was omitted.

### 2.8. Construction of an Expression Vector of nahAcAd and Its Biotransformation Assay

*E. coli* cells containing pBES3, (3-kb EcoRI–SmaI fragment including *nahAcAd* (Figure 2) in pBluescript II KS(+) were cultivated in the presence of 1 mM IPTG in 400 mL LB medium containing 100 μg/mL ampicillin at 37 °C. Harvested cells of the recombinant *E. coli* were suspended in Buffer A and sonicated by Homogenizer subsonic HMO-100 (AGC Techno Glass Co., Ltd., Shizuoka, Japan) for 3 min. Cell debris was removed by centrifugation for 30 min at 18,000× *g*. The supernatant was referred to as the cell-free extract. *E. coli* cells containing pBES3 was cultured in LB medium with appropriate antibiotics. After the overnight cultivation at 37 °C, 1 mM IPTG was added and the cells were cultivated for another 2 h. The cells were harvested, washed and suspended in LB medium at an OD_600_ = 2. Five milliliters of the bacterial suspension was transferred to a test tube and naphthalene was added at a concentration of 1 mM. The tubes were incubated at 37 °C for 2 h. The cell suspension was acidified by the addition of 50 μL concentrated HCl, an equal volume of ethyl acetate was added and mixed for 10 min. After centrifugation at 5000× *g* for 10 min to extract the transformants, the ethyl acetate was concentrated under a stream of nitrogen and trimethylsilylation done using BSTFA (*N*,*O*-bis(trimethylsilyl)trifluoroacetamide) + TMCS (trimethylchlorosilane) (Sylon BFT kit, Sigma-Aldrich Japan Inc., Tokyo, Japan). The derivatized samples were analyzed by gas chromatography (Hewlett-Packard model 6890, Agilent Technologies Japan, Tokyo, Japan), equipped with an HP-5ms capillary column (50 m, 0.2 mm, 0·33 µm, Agilent Technologies Japan, Tokyo, Japan) and a mass selective detector (Hewlett-Packard, model 5972A, Agilent Technologies Japan, Tokyo, Japan). The condition of GC-MS has been described previously [10].

### 2.9. Nucleotide Sequence Accession Number

The genome sequence of *Geoacillus* sp. JF8 is deposited at CP006254- CP006255.

## 3. Results

### 3.1. Determination of N-terminal Amino Acid Sequence of Cis-Naphthalene Dihydrodiol Dehydrogenase

In order to obtain information of N-terminal amino acid sequence of the dehydrogenase, NahB_JF8 was purified from naphthalene-grown cell of *Geobacillus* sp. JF8. The cell extract was subjected to the five step purification procedures (Table 1). We eluted twice for the Phenyl Sepharose column with ammonium sulfate and water because both eluted fractions showed enzymatic activities. The resultant fractions were combined and subjected to the MonoQ column. The purity of NahB_JF8 was assessed by SDS-PAGE analysis, and a single protein was observed at 33 kDa during SDS-PAGE, which was larger than the calculated molecular mass (27 kDa). The N-terminal amino acid sequence of the purified enzyme was determined by Edman degradation to be TKRLENKVAFITGAAGGQGRAAAIVFAREGAKVAVVDVDAKGIEET-ARLVNEAGGE AIAIP XDVSN(E/N)EQVKQAI(I/Q)QTVN.

### 3.2. Cloning and Characterization of *nah* Genes

Using a DNA probe spanning the determined N-terminal region of the purified dehydrogenase, two overlapping DNA fragments, a 6-kb SacI-SmaI fragment and 5-kb EcoRI fragment, were cloned, which were designated as pBSS6 and pBEI5, respectively. By genome walking, a 4-kb BamHI fragment downstream of pBEI5 was cloned and it was designated as pBBm4. A 10-kb DNA fragment containing the flanking region of above *nahB*_JF8 was obtained and their nucleotide sequence was determined. Thirteen open reading frames were identified as shown in Figure 2.

**Figure 3:**
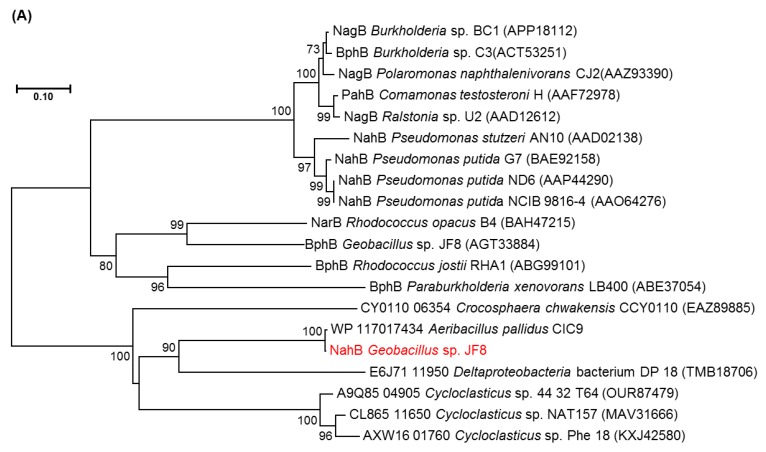

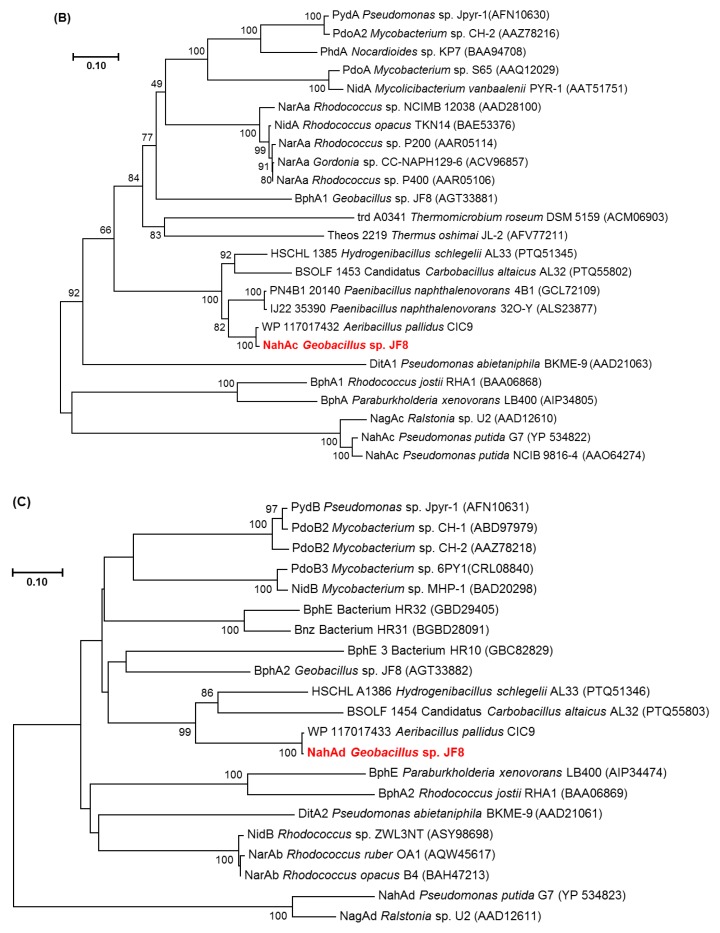
Neighbor-joining tree of amino acid sequences of (**A**) NahAc, (**B**) NahAd, and (**C**) NahB of JF8 (shown in red) with representative homologous proteins in mesophilic- and thermophilic-degraders. Bootstrap values (1000 replications) are shown as percentages at nodes. The tree was reconstructed using MEGA software. Bar, 0.1 substitutions per amino acid position.

The deduced N-terminal amino acid sequence of NahB was MTKRLENKVAFITGAAGGQGR AAAIVFAREGAKVAVVDVDAKGIEETARLVNEAGGEAIAIPCDVSNEEQVKQAIQQTVN, which is identical to the N-terminal amino acid sequence of the purified dehydrogenase from naphthalene grown cell of *Geobacillus* sp. JF8. The amino acid sequence of NahB_JF8 exhibited 99% identity to those of a putative dehydrogenase from *Aeribacillus pallidus* CIC9 (WP_117017434), whereas its activity was not elucidated yet. Those of NahB_JF8 showed low identities with those of mesophilic naphthalene degraders; 46% identity to a 2,5-dichloro-2,5-cyclohexadiene-1,4-diol dehydrogenase from *Crocosphaera chwakensis* CCY0110(EAZ89885), and 31% identity to *cis*-naphthalene dihydrodiol dehydrogenase (NahB) from *Pseudomonas putida* G7 (BAE92158). In a phylogenetic tree of dehydrogenases, NahB_JF8 was not located in the same clade containing *cis*-naphthalene dihydrodiol dehydrogenase, *cis*-biphenyl dihydrodiol dehydrogenase, or *cis*-toluene dihydrodiol dehydrogenase (Figure 3A).

There were two ORFs upstream of *nahB*, and they were designated as *nahAc* and *nahAd*, respectively (Figure 2). Their deduced amino acid sequences exhibited similarity to naphthalene 1,2-dioxygenase α- and β-subunit, respectively. The amino acid sequences of NahAc of strain JF8 showed 98% identity with those of a putative large subunit of aromatic dioxygenase in *Aeribacillus pallidus* CIC9 (WP_117017432). Those showed a 53% identity to a large subunit of naphthalene dioxygenase in *Rhodococcus* sp. P400 (AAR05106) [19]. The conserved amino acid sequences for a [2Fe-2S] Rieske-type cluster were found in 95–118 residues ([2Fe–2S] cluster binding residues, Cys-X-His-X_15-17_-Cys-X_2_-His). Three conserved residues (His, His, Asp) for a catalytic non-heme ion were found in His-222, His-227, and Asp-379 (corresponding to His-208, His-213, and Asp-362 of naphthalene dioxygenase of *P. putida* NCIB 9816). The amino acid sequences of NahAd of strain JF8 showed 99% identity with those of a putative small subunit of aromatic dioxygenase in *Aeribacillus pallidus* CIC9 (WP_117017433). Those showed 47% identity with those of a small subunit of ring-hydroxylating dioxygenase of polycyclic aromatic hydrocarbons in *Mycobacterium* sp. CH-2 (PdoB2) (AAZ78218), and 32% identity with those of a naphthalene 1,2-dioxygenase small subunit from *P. putida* G7 (NahAd) (YP_534823). In a phylogenetic tree based on the amino acid sequences of ring-hydroxylating dioxygenase, NahAc and NahAd of strain JF8 were located in the clades of proteins found in Gram-positive bacteria, respectively (Figure 3B,C). The amino acid sequences of the other ORFs showed similarity with those of *meta*-cleavage product hydrolases (Orf5B), monooxygenase (Orf6B), dehydrogenases (Orf7B), and gentisate dioxygenases (Orf8B) (Figure 2).

### 3.3. Gene Expression Analysis

**Figure 4:**
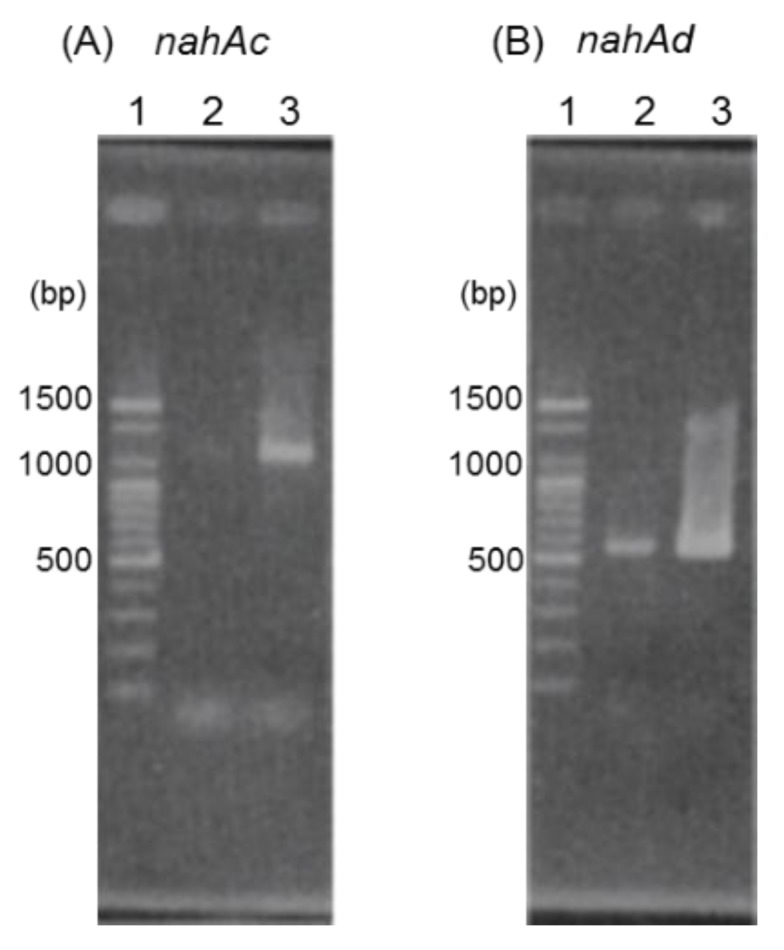
RT-PCR analyses using primer sets designed to amplify *nahAc* (**A**) and *nahAd* (**B**). Lane 1, 100 bp DNA ladder; lane 2, RNA extracted from strain JF8 grown on LB plate was used as template; lane 3, RNA extracted from strain JF8 grown on LB plate supplied with naphthalene as a vapor.

The results of agarose gel electrophoresis of RT-PCR products with RNAs isolated from JF8 grown on LB plate or grown with naphthalene as a vapor were shown in Figure 4. More products of *nahAc* and *nahAd* were detected when the naphthalene was added, suggesting that transcription of *nahAc* and *nahAd* gene were induced by naphthalene.

### 3.4. Biotransformation Assay of *nahAc* and *nahAd* Gene Products

After expressing of *nahAc* and *nahAd* in *E. coli*, naphthalene was added in a reaction mixture. No metabolite was observed from extracts of the reaction mixture.

## 4. Discussion

In the present study, the genes related to naphthalene degradation were isolated and characterized from a thermophilic PCB and naphthalene degrader, *Geobacillus* sp. strain JF8. An enzyme, which dehydrates *cis*-naphthalene dihydrodiol, was successfully purified and a gene encoding the enzyme, NahB, was identified. The genes encoding terminal components of naphthalene dioxygenase, *nahAc* and *nahAd*, were found in the upstream area of *nahB*. Transcriptions of *nahAc* and *nahAd* were induced by naphthalene, indicating that these gene products might be related to naphthalene degradation.

Phylogenetic analysis of NahAc_JF8 and NahAd_JF8 suggested that each of them was classified into a different sub-group from that of large- and small-subunit of PAH-initial dioxygenase found in other Gram-positive bacteria. The group contains hypothetical proteins found in thermophilic bacteria including dibenzofuran- and naphthalene-degrader, *Paenibacillus naphthalenovorans* 4B1 [20]. Similarly, NahB_JF8 was not clustered into a group of *cis*-dihydrodiol dehydrogenases derived from mesophilic PAH degraders. The amino acid sequences of NahB_JF8 showed high identity with those of a thermophilic bacterium, *Aeribacillus pallidus* CIC9, although its function was not elucidated. It should be noted that *Aeribacillus pallidus* was previously *Geobacillus pallidus* [21], and thus, these genes could be conserved in the relatively close genus. These results suggest that the naphthalene-degradation pathway of *Geobacillus* sp. JF8 has evolved through a different route from the previously-known pathways derived from mesophilic bacteria.

In this study, the activity of NahA, a putative terminal dioxygenase of naphthalene, was not detected. This was probably because the NahAc and NahAd were not appropriately expressed in *E. coli*. Another possible reason was that electron transfer systems for the dioxygenase were not supplied in *E. coli*. Genes encoding ferredoxin and its reductase could be located around genes encoding terminal dioxygenase, but the strain JF8 did not contain them near *nahAc* and *nahAd*. Comparing the amino acid sequences of NahAc of strain JF8, NarAa of *Rhodococcus* sp. NCIMB 12038, and NahAc of *Pseudomonas putida* NCIB 9816-4 revealed that the important residues for the enzymatic activity were conserved, which contribute to Rieske-center and active center. In contrast, the residues for electron transfer were not conserved, suggesting that ferredoxin and its reductase might be different from the previously-known ones.

**Figure 5:**
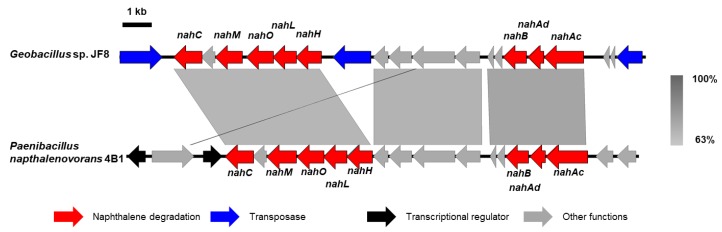
Alignment of nah gene clusters of strain JF8 with that of *Paenibacillus naphthalenovorans* 4B1. Coding sequences of each genome are shown as colored arrows.

In our previous study, we identified 1,2-dihydroxynaphthalene dioxygenase gene (*nahC*) in strain JF8 [12]. Recently, the complete genome sequences of JF8 was determined [19]. The genes responsible for catechol metabolism were found in the upstream of *nahC* from the complete genome sequence of JF8 [22] (Figure 5, *nahHLOM*). A putative gene encoding 2-hydroxymuconic semialdehyde hydrolase (NahN), which convert 2-hydroxymuconic semialdehyde to 4-oxopent-4-enoate, was found downstream of *nahB* (Figure 5). The amino acid sequences of the product showed a 75% identity with previously-known 2-hydroxymuconic semialdehyde hydrolase of *Hydrogenibacillus schlegelii* AL33 (PTQ51003), 32% to NahN of *Pseudomonas stutzeri* AN10 (AAD02150). It should be noted that a *nah* gene cluster of JF8 was found in a moderate thermophilic dibenzofuran- and naphthalene-degrader, *Paenibacillus naphthalenovorans* 4B1 (Figure 5). Gene order and putative amino acid sequence are highly conserved between strains JF8 and 4B1, whose identities were 66–86%. Currently, we could not find putative genes encoding NahD, NahE, and NahF, which degrade meta-cleavage product to salicylate (Figure 1) in the genome sequence of strain JF8. On the other hand, putative gentisate dioxygenase was found (Orf8B, Figure 2) in the downstream area of *nahAB* genes, suggesting that JF8 could metabolize salicylate via gentisate.

Metabolic pathways, enzymes, and genes involved in PAH degradation were extensively studied in Gram-negative bacteria [23,24,25]. Genetic and biochemical analyses about PAH-degrader of Gram-positive bacteria were also reported including thermophilic microorganisms [20,26,27,28,29,30,31,32,33]. These facts imply that Gram-positive bacteria can play more important roles in the degradation of high molecular PAHs including biphenyl [34,35,36,37,38,39,40,41]. Further investigation including molecular analysis will provide us more clear information on naphthalene degradation including a lower pathway of thermophilic bacteria.

## Figures and Tables

**Table 1:** Purification of *cis*-naphthalene dihydrodiol dehydrogenase (NahB).

Step	Volume	Total Protein	Total Activity	Specific Activity	Yield
	(mL)	(mg)	(U)	(U/mg)	(%)
Cell-free extract	150	536	122	0.228	100
DEAE Toyopearl 650M	148	58	21.9	0.378	18
Phenyl Sepharose ((NH_4_)_2_SO_4_-eluted)	48	4	7.8	1.95	6.4
Phenyl Sepharose (H_2_O-eluted)	11	0.8	0.30	0.375	0.25
MonoQ	1.0	0.075	0.091	1.21	0.074
